# Emergent gastric remnant volvulus in a post-OAGB patient with massive hiatal herniation: a life-threatening presentation in an asymptomatic course

**DOI:** 10.1093/jscr/rjaf692

**Published:** 2025-09-09

**Authors:** Yosor Fiesal, Leonid Drober, Sa’d Sayida, Ahmad Assalia

**Affiliations:** Department of General Surgery, Rambam Medical Center and the Bruce Rappaport Faculty of Medicine, Technion – Institute of Technology, 8 HaAliya Hashniya Street, Haifa, Israel; Department of General Surgery, Rambam Medical Center and the Bruce Rappaport Faculty of Medicine, Technion – Institute of Technology, 8 HaAliya Hashniya Street, Haifa, Israel; Department of General Surgery, Rambam Medical Center and the Bruce Rappaport Faculty of Medicine, Technion – Institute of Technology, 8 HaAliya Hashniya Street, Haifa, Israel; Department of General Surgery, Rambam Medical Center and the Bruce Rappaport Faculty of Medicine, Technion – Institute of Technology, 8 HaAliya Hashniya Street, Haifa, Israel

**Keywords:** mini-gastric bypass, gastric remnant volvulus, hiatal hernia

## Abstract

One anastomosis gastric bypass (OAGB) is a widely used bariatric procedure with favorable outcomes and a relatively low complication rate. However, both early and late postoperative complications can occur, including rare but serious events such as gastric remnant volvulus, particularly in the presence of massive hiatal herniation. The altered anatomy following OAGB can make the diagnosis of such complications challenging, especially when clinical presentations are atypical or silent. We report the case of a 67-year-old woman with a history of OAGB and prior hiatal hernia repair who presented nearly 4 years postoperatively with isolated elevations in cholestatic liver enzymes but no abdominal symptoms. Imaging revealed a massive hiatal hernia containing the gastric pouch, gastrojejunal anastomosis, remnant stomach, and a portion of the pancreas, with evidence of partial gastric remnant volvulus and duodenal obstruction. The patient developed acute gastrointestinal bleeding during hospitalization, prompting urgent laparoscopic intervention. Intraoperative findings confirmed a large hiatal defect with herniation and rotation of the gastric remnant. Surgical management included reduction of herniated organs, gastrotomy for decompression, hiatal repair, and resection of the remnant stomach. The patient recovered uneventfully. This case underscores the importance of maintaining a high index of suspicion for rare but life-threatening complications such as gastric remnant volvulus in post-OAGB patients, even in the absence of classic symptoms. Timely imaging and prompt surgical intervention are essential for favorable outcomes. Ongoing, individualized follow-up and multidisciplinary care are crucial for early detection and management of late bariatric surgery complications.

## Introduction

Obesity is a global health concern, and bariatric surgery has become an increasingly common and effective intervention for weight loss and the management of obesity-related comorbidities. One anastomosis gastric bypass (OAGB), also known as mini-gastric bypass, is a popular bariatric procedure due to its relative technical simplicity, favorable outcomes, and lower complication rates compared to other techniques. Despite its benefits, OAGB does carry certain risks, and patients may experience both early and late postoperative complications [1].

Late complications are more frequent and include marginal ulceration, malnutrition, anemia, bile reflux, and gastroesophageal reflux disease (GERD) [2]. Internal hernias (including Petersen’s hernia) are rare but recognized, and can lead to serious sequelae such as bowel obstruction or, in rare cases, gastric remnant perforation [3]. Gastric remnant volvulus is not commonly reported in the literature after OAGB, but the presence of a massive hiatal hernia or altered anatomy from prior bariatric or foregut surgery may predispose patients to this life-threatening complication. In such cases, volvulus can present acutely with obstruction, ischemia, or perforation, requiring prompt diagnosis and surgical intervention. In the context of OAGB, the remnant stomach is typically excluded from the alimentary tract, making clinical presentations of volvulus atypical and diagnosis more challenging [4, 5].

Hiatal herniation is a recognized but uncommon complication after bariatric surgery, including OAGB, and its clinical significance increases when it coexists with gastric remnant volvulus, as both conditions can lead to life-threatening presentations. The altered anatomy after OAGB, particularly with a long gastric pouch or prior hiatal hernia, predisposes patients to intrathoracic migration of the gastric pouch or remnant, which can result in volvulus or obstruction [5–7]. This underscores the importance of maintaining a high index of suspicion in post-bariatric surgery patients presenting with nonspecific or acute abdominal symptoms.

We present a rare case of acute gastric remnant volvulus in a previously asymptomatic patient with a history of OAGB and massive hiatal herniation. This case underscores the diagnostic complexities and emphasizes the importance of timely recognition and management of this uncommon yet serious complication.

## Case report

A 67-year-old woman with a medical history significant for type 2 diabetes mellitus, hypertension, and hyperlipidemia underwent OAGB in 2020, along with a posterior primary repair of a small hiatal hernia using three Ethibond sutures. At the time of surgery, her weight was 95 kg, corresponding to a BMI of 37. She subsequently experienced consistent weight loss, ultimately achieving a BMI of 23.

**Figure 1:**
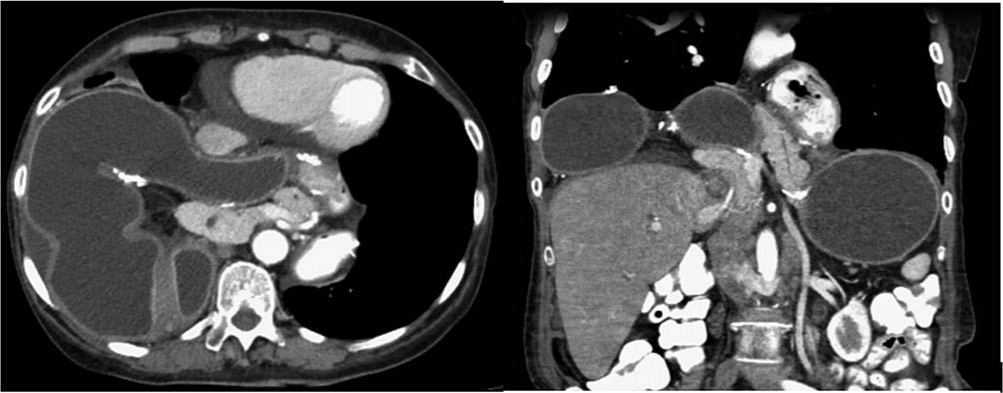
CT scan—the gastric pouch, gastrojejunal anastomosis, remnant stomach, and a portion of the pancreas were located within the thoracic cavity above the diaphragm.

Nearly 4 years after her surgery, the patient presented to her surgeon’s clinic without any symptoms, but routine laboratory tests revealed isolated elevations in cholestatic liver enzymes. An abdominal ultrasound identified gallbladder sludge and dilation of the common bile duct to 0.9 mm, with no evidence of cholecystitis or gallstones. Additionally, a large right supradiaphragmatic fluid collection was observed. Further evaluation with computed tomography (CT) demonstrated a massive hiatal hernia containing the gastric pouch, gastrojejunal anastomosis, gastric remnant, and a portion of the pancreas (Fig. 1). The imaging also showed signs consistent with partial volvulus of the gastric remnant and associated duodenal obstruction.

Despite being asymptomatic at admission, the patient was hospitalized for preoperative evaluation. On the second day of her hospital stay, she began experiencing nausea and a reduced appetite, which was soon followed by a significant episode of massive coffee-ground vomiting. Although her vital signs remained stable and her physical examination was unremarkable, her hemoglobin level dropped markedly from 14.6 to 10.7 g/dl.

**Figure 2:**
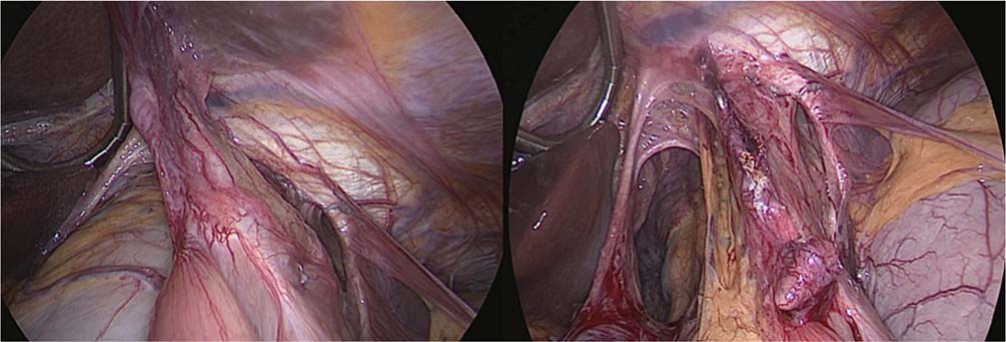
Intraoperative view—large hiatal defect before and after reduction herniated viscera.

An urgent laparoscopic exploration was performed, revealing a large hiatal defect with exposure of the posterior mediastinum and aorta. The proximal portion of the gastric remnant remained within the abdominal cavity, while the distal remnant and duodenum had herniated into the thoracic cavity and exhibited rotation. The remnant stomach was found to be significantly distended and edematous, containing ~3 l of coffee-ground fluid. Additionally, the entire gastric pouch and gastrojejunal anastomosis were discovered herniated within the chest (Fig. 2).

**Figure 3:**
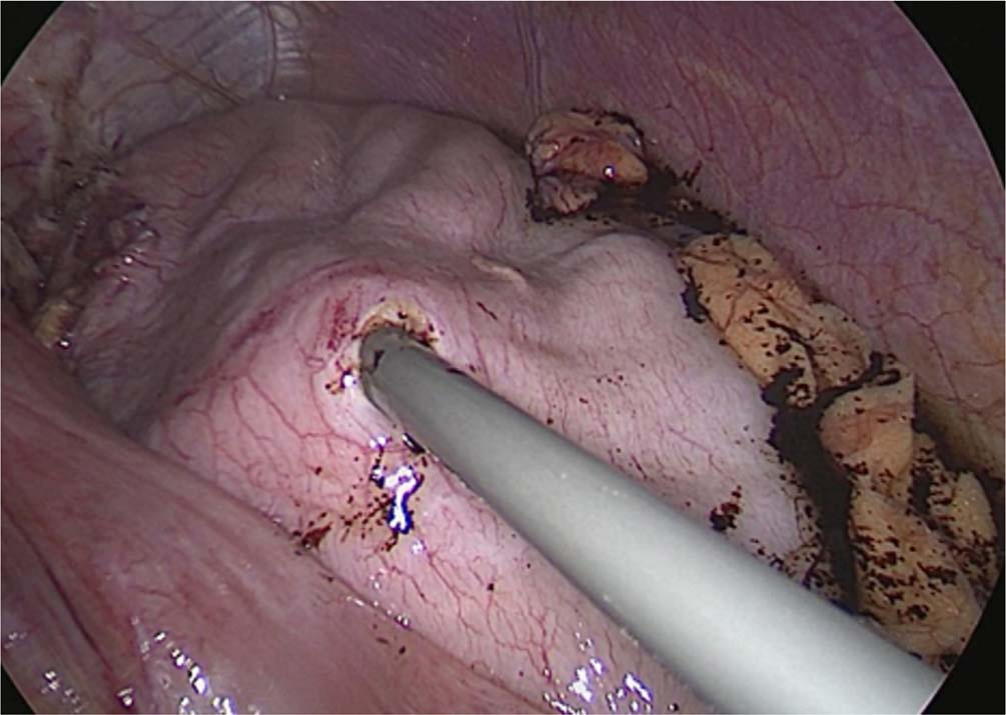
Intraoperative view—gastrotomy and suction of remnant content.

**Figure 4:**
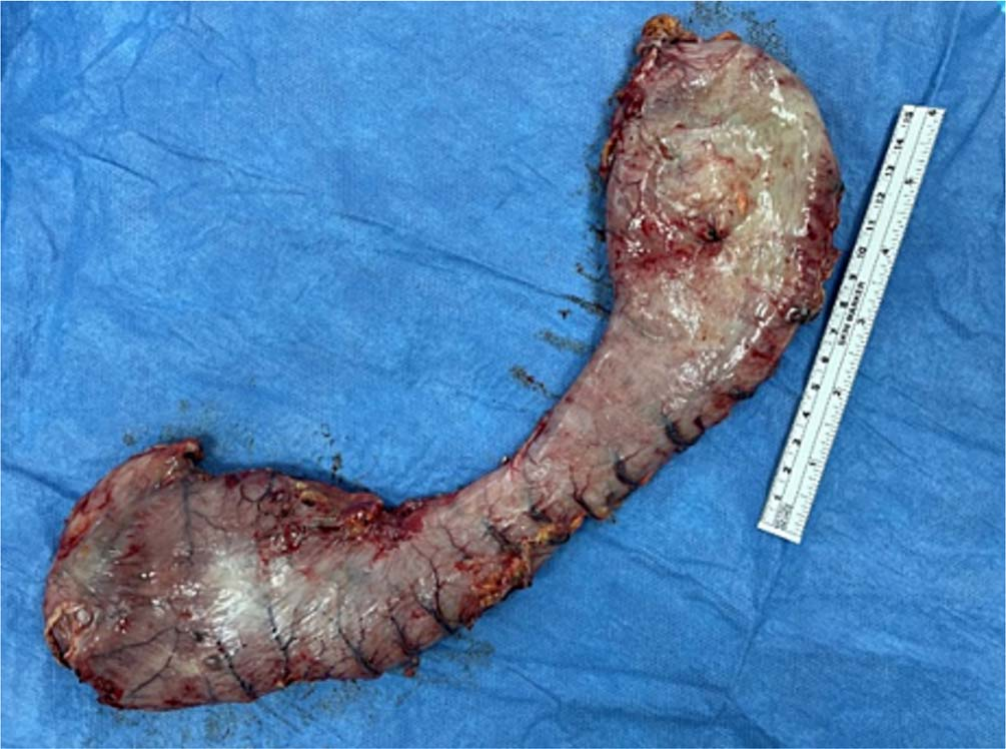
Resected gastric remnant.

Surgical management involved reducing all herniated abdominal organs from the thoracic cavity. To address the distention of the gastric remnant, a gastrotomy was performed to evacuate the accumulated fluid (Fig. 3). This was followed by a primary hiatal repair using an anterior approach, and resection of the remnant stomach up to the level of the duodenum (Fig. 4).

The patient’s postoperative recovery was smooth and without complications, and she was discharged in good condition on the second day after surgery.

## Discussion

We report a rare but potentially life-threatening case of acute gastric remnant volvulus occurring years after an OAGB, compounded by a massive hiatal hernia. Although hiatal herniation is recognized as an infrequent yet significant late complication following OAGB and other bariatric procedures, its coexistence with gastric volvulus markedly increases the diagnostic complexity. Both conditions can present with nonspecific symptoms—such as abdominal pain, vomiting, and signs of obstruction or ischemia—making timely diagnosis challenging.

Gastric remnant volvulus is seldom reported following OAGB, likely due to the remnant stomach’s exclusion from the alimentary tract, which results in atypical or delayed clinical manifestations. This diagnostic difficulty is further compounded by the often subtle or absent symptoms, as seen in our patient, until catastrophic complications—such as ischemia, perforation, or massive gastrointestinal bleeding—occur. The insidious onset observed in this case highlights the importance of vigilance during follow-up, even in asymptomatic patients or those exhibiting nonspecific biochemical abnormalities, such as isolated elevations in cholestatic enzymes.

The true incidence of acute gastric remnant volvulus post-OAGB remains unknown and is poorly represented in the current literature. Large clinical series and multi-institutional surveys rarely identify this condition as a recognized late complication, in contrast to well-documented issues like marginal ulceration, malnutrition, anemia, bile reflux, and internal hernias [8, 9]. Most published accounts of late gastric remnant pathology focus on internal hernias [10], especially Petersen’s hernia, which can predispose to remnant complications such as perforation or, more rarely, volvulus. This case illustrates how previous surgical interventions—particularly posterior hiatal hernia repair—may disrupt normal intra-abdominal dynamics, facilitating organ migration and rotation. In such cases, cross-sectional imaging, notably CT, is indispensable, especially when ultrasound findings are inconclusive.

Prompt recognition and urgent laparoscopic management are essential to prevent dire outcomes such as gastric ischemia, perforation, and sepsis [11]. This is especially crucial when massive hiatal hernia and prior surgical manipulation are present. Delayed diagnosis is associated with increased morbidity and mortality, underscored by the high fatality rates in cases complicated by necrosis or perforation. Surgical treatment should encompass both hernia repair and decompression or resection of compromised gastric tissue to reestablish normal anatomy and prevent recurrence [12].

## Conclusion

This case highlights the rare but potentially fatal complication of acute gastric remnant volvulus in a patient with a history of OAGB and massive hiatal herniation. The atypical and often silent clinical presentation, as seen in our patient, underscores the importance of maintaining a high index of suspicion for late complications in post-bariatric surgery patients, even in the absence of classic symptoms. Prompt recognition through appropriate imaging and timely surgical intervention are critical to prevent severe morbidity and mortality. This report emphasizes the need for ongoing, individualized follow-up and multidisciplinary management in bariatric patients, particularly those with altered anatomy or prior hernia repairs. Increased awareness of this rare entity among clinicians can facilitate earlier diagnosis and improve patient outcomes in similar scenarios.
